# Intraosseous Versus Intravenous Vascular Access in Out-of-Hospital Cardiac Arrest: A Systematic Review and Meta-Analysis of Randomized Controlled Trials

**DOI:** 10.3390/medsci13020078

**Published:** 2025-06-14

**Authors:** Alhareth Alsagban, Omar Saab, Hasan Al-Obaidi, Marwah Algodi, Amy Yu, Mohamed Abuelazm, Chad Hochberg

**Affiliations:** 1 Department of Medicine, Johns Hopkins University, Baltimore, MD 21287, USA; 2 Department of Medicine, University of Texas at Houston, Houston, TX 77082, USA; 3 Jamaica Hospital Medical Center, Department of Medicine, Queens, NY 11418, USA; 4 Department of Medicine, College of Medicine, University of Baghdad, Baghdad 00964, Iraq; 5 Department of Medicine, Faculty of Medicine, Tanta University, Tanta 31111, Egypt

**Keywords:** resuscitation, epinephrine, CPR, emergency, ambulance

## Abstract

**Background.** Establishing prompt vascular access facilitates resuscitation for out-of-hospital cardiac arrest (OHCA). While intraosseous access may decrease the time to vascular access, the impact on clinical outcomes in OHCA is unclear. Therefore, we aim to compare the effect of intraosseous (IO) versus intravenous (IV) vascular access on clinical outcomes after OHCA resuscitation. **Methods.** A systematic review and meta-analysis were performed to synthesize evidence from randomized controlled trials (RCTs) obtained from PubMed, CENTRAL, Scopus, and Web of Science until January 2025. Using Stata MP v. 17, we used the fixed-effects model to report dichotomous outcomes using the risk ratio (RR) and continuous outcomes using the mean difference (MD) with a 95% confidence interval (CI). PROSPERO ID: CRD42024627354. **Results.** Four RCTs and 9475 patients were included. There was no difference between both groups regarding the prehospital return of spontaneous circulation (ROSC) (RR: 0.97, 95% CI [0.91, 1.03], *p* = 0.33), maintained ROSC (RR: 0.94, 95% CI [0.87, 1.01], *p* = 0.09), survival to discharge (RR: 1.03 with 95% CI [0.88, 1.21], *p* = 0.71), 30-day survival (RR: 0.98, 95% CI [0.82, 1.17], *p* = 0.79), or favorable neurological recovery (RR: 1.07, 95% CI [0.90, 1.29], *p* = 0.44). However, IO access significantly increased first-attempt access (RR: 1.24, 95% CI [1.19, 1.29], *p* < 0.001), decreased time to vascular access (MD: −0.24 min with 95% CI [−0.48, −0.01], *p* = 0.04), and decreased time to drug administration (MD: −0.38, 95% CI [−0.66, −0.10], *p* = 0.01). **Conclusions.** IO and IV vascular accesses showed similar clinical outcomes in OHCA patients, with no difference in ROSC, survival, or neurological recovery. Still, IO access showed a better procedural outcome with increased first-attempt success rates, faster access, and faster drug administration.

## 1. Introduction

Globally, out-of-hospital cardiac arrest (OHCA) represents a leading cause of mortality, with incidence estimates ranging from 30 to 97 cases per 100,000 person-years for emergency medical service (EMS)-managed OHCA [1,2,3,4]. Survival to hospital discharge in OHCA patients remains dismal at about 8%, even with improved community response to OHCA, such as bystander cardiopulmonary resuscitation (CPR) and public access defibrillation [3,4,5,6,7]. A cornerstone of OHCA EMS-managed resuscitation is prompt administration of chest compressions, defibrillation (if appropriate), and drug therapy (primarily epinephrine) [8,9]. While the intraosseous (IO) vs. intravenous (IV) route could facilitate faster and durable vascular access [2], its effect on clinical outcomes has only recently been examined in randomized clinical trials (RCTs) [10,11,12,13].

Current resuscitation guidelines recommend prioritizing IV access for drug administration during OHCA while designating the IO route as an alternative when intravenous access is not feasible [8,9]. However, this recommendation is based on a small RCT [12] showing similar procedural outcomes. Several observational studies have also been performed in this area, providing conflicting evidence. They are likely to suffer from indication bias (reflecting IO access placed after failed IV attempts) [14,15]. In practice, IO access has been increasing in clinical practice [2] as technologies to facilitate the ease of access have improved. In response to uncertainty over the optimal route, two large RCTs studying this question were recently published [10,13], providing no evidence of significant clinical effects on their primary outcomes (30-day survival and sustained return of spontaneous circulation (ROSC), respectively). However, secondary analysis suggested the possibility of variable outcomes [10,11,13].

Three major RCTs were recently published, providing a direct parallel head-to-head comparison of IO and IV vascular access during OHCA resuscitation [10,11,13]. Therefore, this systematic review and meta-analysis aims to compare the effect of IO versus IV vascular access on clinical outcomes after OHCA resuscitation, providing clear evidence on which vascular access should be recommended in future resuscitation guidelines.

## 2. Methodology

### 2.1. Protocol Registration

We registered this systematic review with the International Prospective Register of Systematic Reviews (PROSPERO) at CRD42024627354 before the review process. This study and manuscript follow the guidance in the Preferred Reporting Items for Systematic Reviews and Meta-Analyses (PRISMA) statement [16] and the Cochrane Handbook for Systematic Reviews of Interventions [17].

### 2.2. Data Sources and Search Strategy

A systematic electronic search was conducted on 09th January 2025 by AA and OS using the following databases: PubMed (MEDLINE), Web of Science (WOS), Scopus, and Cochrane CENTRAL. The search strategy included the following search keywords: “(Intraosseous OR “Intra-osseous”) AND (intravenous OR IV) AND (“heart arrest*” OR “cardiac arrest*” OR “cardiopulmonary arrest*” OR “sudden cardiac death” OR SCD OR “OHCA” OR “ventricular tachycardia” OR “ventricular fibrillation” OR “ventricular arrythmia*” OR “pulseless electrical activity” OR PEA OR arrest*)”. The search was conducted without limits or filters, apart from Scopus, for which the scope was delimited to titles, abstracts, and keywords. Further details on each database’s search terms and results are shown in Table S1. A thorough manual search of the trial list references was undertaken to ensure a complete review and avoid any eligible records being excluded.

### 2.3. Eligibility Criteria

We included (1) RCTs that enrolled (2) adults with OHCA from any cause, and (3) compared IO to IV access. IO access could include any site (e.g., humeral, tibial, sternal).

#### Outcomes

Our primary outcome of interest was ROSC, either prehospital (a pre-requisite for subsequent survival) or sustained ROSC. Secondary outcomes were survival to hospital discharge, survival to day 30, favorable neurological recovery (defined as Modified Rankin Scale (mRS) 0–3 or Cerebral Performance Category (CPC) 0–2), and unfavorable neurological recovery (defined as mRS 4–6 or CPC 3–5). We additionally compared procedural outcomes with a particular interest in first-attempt vascular access success, time between emergency medical service (EMS) arrival and vascular access, and time between EMS arrival and drug administration.

### 2.4. Study Selection

The screening process was conducted using the Covidence online tool to manage references [CITE]. Independent reviewers (AA, AY, and MA) carried out a thorough screening process. After eliminating duplicate entries, each retrieved record underwent a two-stage review process independently by two reviewers. This involved initial title and abstract screening followed by full-text evaluation for potentially eligible records. If the two reviewer ratings of relevance were discrepant, these were resolved in discussion between reviewers.

### 2.5. Data Extraction

Once the full text of eligible studies was obtained, an initial data extraction step was performed to create a data collection sheet using Excel. This data sheet was organized into three main parts, the first focused on key details of the studies: the primary author’s name, the publication year, the country of origin, the research design, the number of participating centers, the overall sample size, specifics of treatment protocols, principal conditions for inclusion, the main outcome measured, how sustained ROSC was defined, and the length of the follow-up period; the second focused on baseline characteristics of the included participants (number of patients in each group, age, gender, bystander cardiopulmonary resuscitation (CPR), OHCA cause, and OHCA location); and the third contained the outcome data as previously described.

Data extraction was independently performed by two reviewers (AA and HA), with discrepancies resolved through discussion and consensus with a senior author. The extraction of dichotomous outcome variables employed event and total formats; continuous outcome variables were extracted with the mean and standard deviation. Data reported as median and interquartile range were converted to mean and standard deviation using the formulas of Wan et al. [18].

### 2.6. Risk of Bias and Certainty of Evidence

The methodological quality of the included RCTs was appraised for risk of bias using the updated RoB 2 tool from the Cochrane Collaboration [19]. This appraisal involved two independent reviewers (AA and CH) who evaluated each trial across domains including selection, performance, reporting, and attrition biases, plus overall bias; disagreements were resolved by reaching a consensus. Furthermore, the Grading of Recommendations Assessment, Development, and Evaluation (GRADE) framework was applied to ascertain the certainty of the evidence [20,21]. This process considered several factors, such as inconsistency, imprecision, indirectness, publication bias, and the risk of bias associated with the studies. Each of these elements underwent a meticulous evaluation, with all judgements explicitly justified and recorded. Divergent assessments among the reviewers were reconciled through team discussion.

### 2.7. Statistical Analysis

Statistical analysis was performed using Stata MP version 17 (StataCorps, Austin, TX, USA). Pooled risk ratios (RRs) were determined for dichotomous outcomes, and mean differences (MDs) were calculated for continuous outcomes; 95% confidence intervals (CIs) were reported for these measures. The primary analytical method was a fixed-effects model, though a random-effects model was adopted if considerable heterogeneity was detected. Statistical heterogeneity among the study results was assessed using the chi-square test alongside the I-squared (I^2^) statistic. Publication bias was not assessed because fewer than 10 RCTs were included in the review [22].

## 3. Results

### 3.1. Search Results and Study Selection

**Figure 1:**
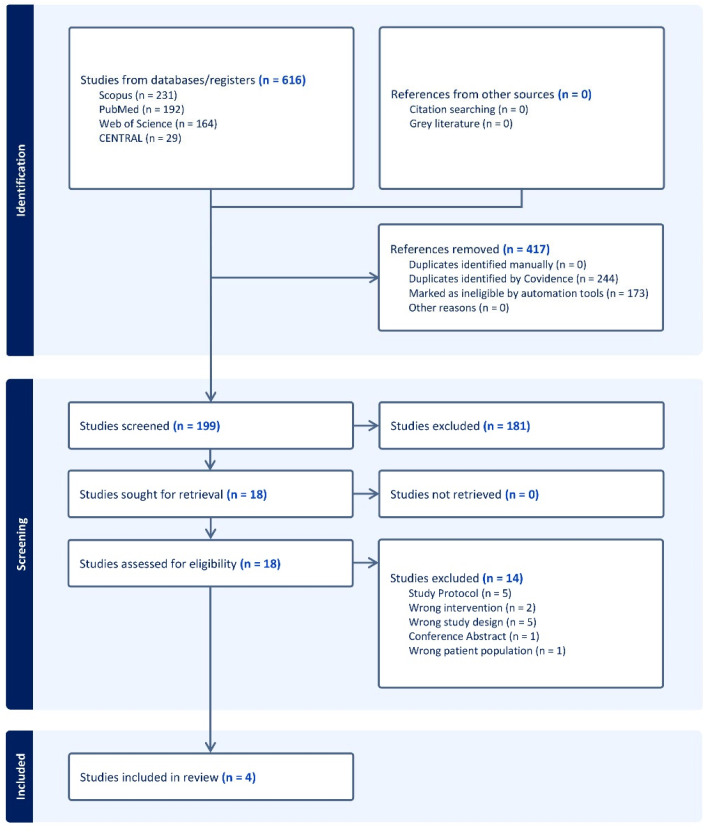
PRISMA flow chart of the screening process.

We identified 616 studies using the search strategy. Of these, 18 full-text articles were identified for further review after removing 417 irrelevant records and 181 studies that did not meet the inclusion criteria. Fourteen were found to be irrelevant and excluded, leaving four studies to be included in qualitative and quantitative assessments (Figure 1).

### 3.2. Characteristics of Included Studies

Four RCTs enrolling a total of 9475 patients (4627 randomized to IO and 4848 to IV) were included [10,11,12,13]. Of these trials, all except Couper et al. excluded traumatic OHCA [10]. One trial assessed outcomes related to the timing and success of vascular access but did not assess patient outcomes and was not included in the primary ROSC or secondary mortality-based analyses [12]. Further summary characteristics of the included trials are presented in Table 1. Key baseline characteristics of the included patients are presented in Table 2.

### 3.3. Risk of Bias and Certainty of Evidence

**Figure 2:**
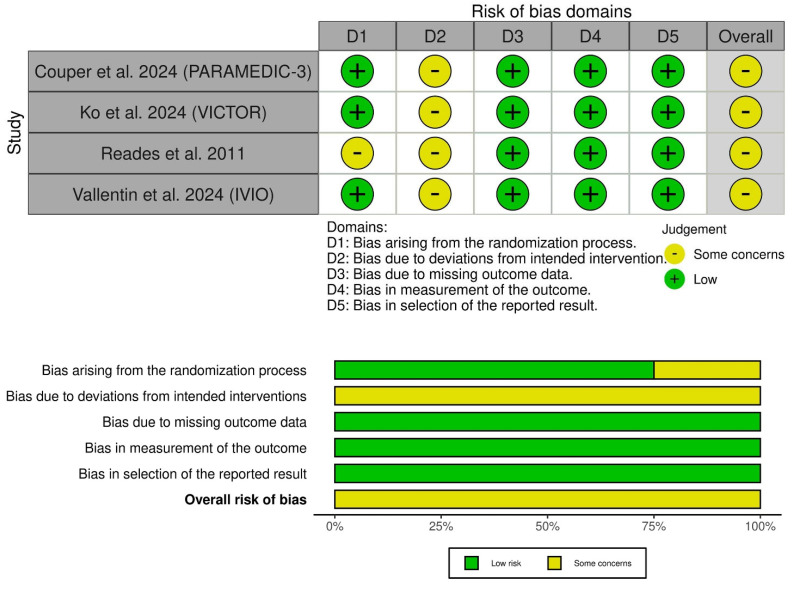
Quality assessment of risk of bias in the included trials. The upper panel presents a schematic representation of risks (low = Green; unclear = yellow; and high = red) for specific types of biases of the studies in the review. The lower panel presents risks (low = Green; unclear = yellow; and high = red) for the subtypes of biases of the combination of studies included in this review [10,11,12,13].

All included trials had some concerns of bias (Figure 2). This was mainly because of the open-label nature of interventions, leading to a risk of performance bias. Also, Reades et al. had some concerns about selection bias due to the lack of information on the randomization process [12]. The GRADE evidence profile illustrates the certainty of evidence (Table 3).

### 3.4. Primary Outcome: ROSC

**Figure 3:**
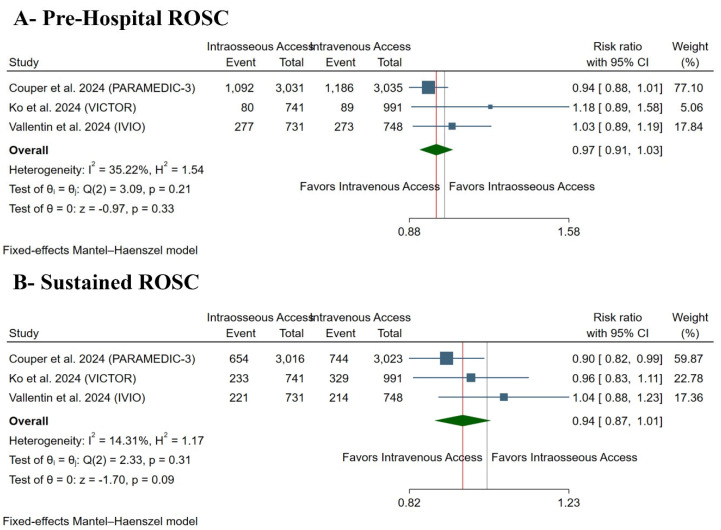
Forest plot of the primary outcomes. CI: confidence interval [10,11,13].

There was no difference between both groups regarding prehospital ROSC in IO vs. IV groups (RR: 0.97 with 95% CI [0.91, 1.03], *p* = 0.33) (Figure 3A) and maintained ROSC (RR: 0.94 with 95% CI [0.87, 1.01], *p* = 0.09) (Figure 3B). Pooled studies were homogenous in prehospital ROSC (I^2^ = 35%, *p* = 0.21) and maintained ROSC (I^2^ = 14%, *p* = 0.31).

### 3.5. Secondary Outcomes

#### 3.5.1. Secondary Clinical Outcomes

**Figure 4:**
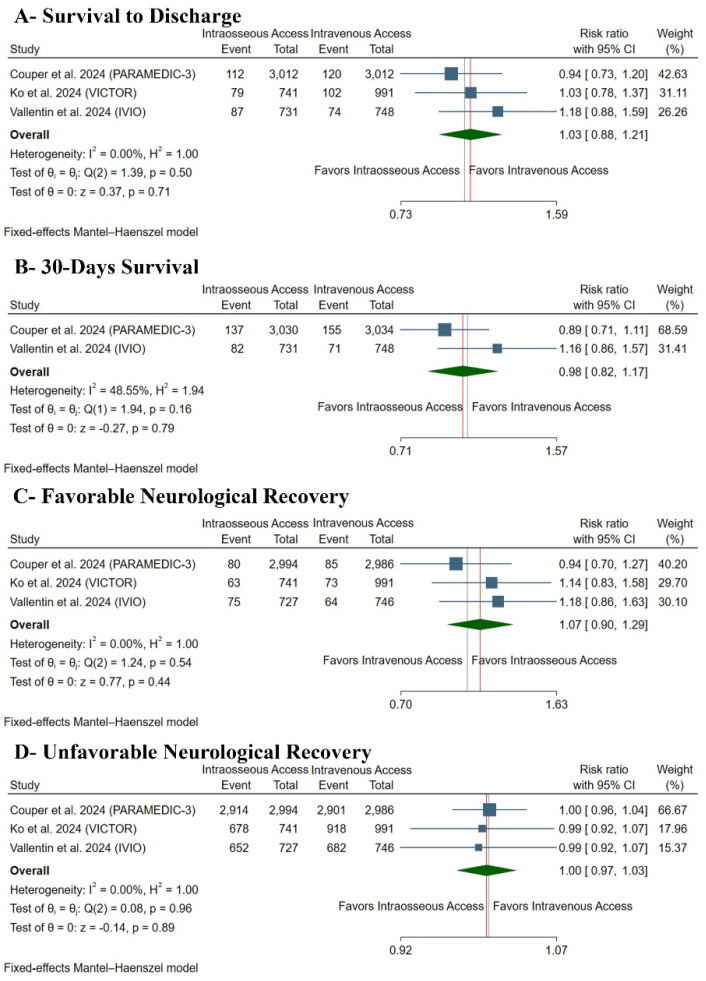
Forest plot of the secondary clinical outcomes. CI: confidence interval [10,11,13].

There was no difference between both groups regarding survival to discharge (RR: 1.03 with 95% CI [0.88, 1.21], *p* = 0.71) (Figure 4A), 30-day survival (RR: 0.98 with 95% CI [0.82, 1.17], *p* = 0.79) (Figure 4B), favorable neurological recovery (RR: 1.07 with 95% CI [0.90, 1.29], *p* = 0.44) (Figure 4C), or unfavorable neurological recovery (RR: 1.00 with 95% CI [0.97, 1.03], *p* = 0.89) (Figure 4D). Pooled studies were homogenous in survival to discharge (I^2^ = 0%, *p* = 0.50), 30-day survival (I^2^ = 48%, *p* = 0.46), favorable neurological recovery (I^2^ = 0%, *p* = 0.54), and unfavorable neurological recovery (I^2^ = 0%, *p* = 0.96).

#### 3.5.2. Secondary Procedural Outcomes

**Figure 5:**
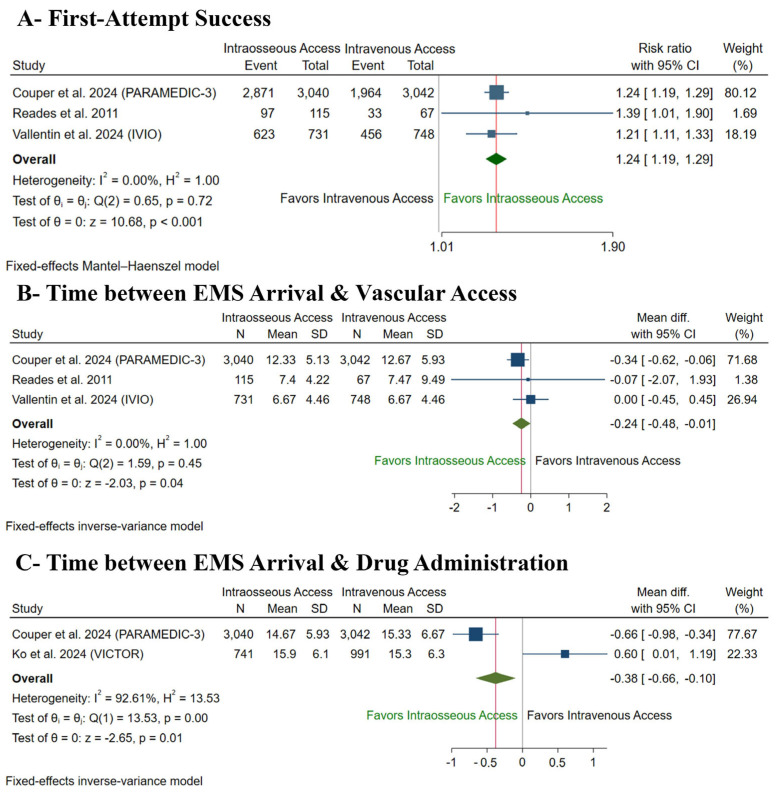
Forest plot of the secondary procedural outcomes. CI: confidence interval [10,12,13].

IO access was significantly associated with increased first-attempt access (RR: 1.24 with 95% CI [1.19, 1.29], *p* < 0.001) (Figure 5A), decreased time from EMS arrival to vascular access (MD: −0.24 min with 95% CI [−0.48, −0.01], *p* = 0.04) (Figure 5B), and decreased time from EMS arrival to drug administration (MD: −0.38 with 95% CI [−0.66, −0.10], *p* = 0.01) (Figure 5C) compared to IV access. Pooled studies were homogenous in first-attempt access (I^2^ = 0%, *p* = 0.72) and time from EMS arrival to vascular access (I^2^ = 0%, *p* = 0.45); however, they were heterogenous in time from EMS arrival to drug administration (I^2^ = 92%, *p* < 0.001), and sensitivity analysis was not applicable as only two studies were pooled.

## 4. Discussion

After pooling data from four RCTs and 9475 patients, IO access did not significantly improve clinical outcomes in OHCA patients, including prehospital ROSC, maintained ROSC, survival, and neurological recovery, compared to IV access. This is despite IO access significantly increasing first-attempt vascular access success, achieving both access and drug administration more quickly.

A previous systematic review of observational studies reported different findings, favoring IV access with low certainty of evidence [23]. Another more recent meta-analysis of observational studies reported similar findings, with IV access demonstrating significantly increased rates of ROSC, survival to discharge, and favorable neurological recovery [23]. Neither review [23,24] included the most recent major RCTs [10,11,13]; therefore, the reliability of their findings is not strong. Observational studies are highly liable to confounding factors, selection bias, and resuscitation time bias. In observational cardiac arrest studies, resuscitation time bias occurs when the duration of resuscitation efforts can influence the likelihood of receiving a particular treatment or intervention, leading to misleading results, as longer resuscitation times are often associated with worse outcomes, thereby skewing the perceived effect of the intervention [25].

An IO-first approach was hypothesized to expedite epinephrine delivery, thus leading to higher rates of ROSC and potentially translating to improved survival outcomes. Decreasing the time to ROSC could also prevent further hypoxic–ischemic damage, the leading cause of death and neurological morbidity following cardiac arrest [26]. This hypothesis is supported by prior research demonstrating expedited drug administration in patients when initial vascular access is established via IO, especially the proximal tibia [12,27]. Our findings, which synthesize four high-quality RCTs, support that IO placement can reduce the time needed for vascular access. Yet, consistent with the conclusions of the individual trials, our meta-analysis showed no clinically or statistically essential differences in survival outcomes.

We hypothesize several factors that could contribute to the lack of better outcomes despite shorter times for drug delivery. First, IO cannulation may be misplaced, resulting in compromised drug bioavailability. While this variable remained unassessed in our analysis, prior research indicates a susceptibility to mispositioning and displacement with IO cannulas [12,28]. Second, IO administration may result in lower peak drug concentrations and a longer time to peak concentration compared to IV administration, even with successful cannulation [10]. Animal studies indicate that IO drug administration via the proximal humerus may achieve peak concentration more rapidly than peripheral OV administration, while proximal tibial administration may be slower [2]. Nevertheless, the potential benefit of the humeral site over the tibial site may be counteracted by decreased success rates, increased dislodgement, and prolonged placement times [12]. Third, compared to IV administration, IO administration of lipophilic drugs, including amiodarone, results in less effective delivery to the systemic circulation [29].

Moreover, IO insertion procedures carry significant risks that should be considered. IO may be complicated by cannula malposition, extravasation, fracture, soft tissue infection, fat embolism, growth plate injury, compartment syndrome, or osteomyelitis [30,31,32,33]. While the reported incidence of significant complications is generally low [2], the comprehensive safety profile of IO access is challenging to interpret due to the potential confinement of detailed follow-up assessments for complications to survivors only [2]. Also, even among survivors, complications such as fat embolism may be unidentified [2]. This highlights the need to investigate the safety profile of IO access further.

Alilou et al. published a systematic review and meta-analysis, which also exclusively synthesized data from the same four recent RCTs [34]. Their findings closely mirror ours, concluding that there were no significant differences between IO and IV access for the outcomes of survival or favorable neurological outcome. Similarly to our results, they also reported a numerically lower, though not statistically significant, rate of ROSC with IO access and confirmed the procedural benefits of IO access, including a significantly higher first-attempt success rate and a faster time to vascular access [34]. Our review further extends these procedural findings by specifically analyzing the time to drug administration, which was also significantly shorter with IO access. The consistency of these two high-quality meta-analyses confirms that while IO access may be more convenient, it offers no clinical advantage in terms of survival or neurological outcomes compared to IV access for OHCA.

Another recent meta-analysis by Rath et al. investigated this topic but reached different conclusions, reporting a clear superiority for IV access over IO in terms of ROSC, survival at admission, discharge, 30-day survival, and favorable neurological outcomes [35]. A critical methodological distinction likely explains this conflict: Rath et al. included data from 19 studies, comprising not only 3 RCTs but also 16 cohort studies, which accounted for the vast majority of their 239,486 patients [35]. As discussed previously in our manuscript and supported by Alilou et al. [34], observational studies in OHCA resuscitation are highly susceptible to several types of biases, where IO access may be disproportionately utilized in patients with more critical conditions or after failed IV attempts. The substantial weight of observational data in the analysis by Rath et al. [35] likely influenced their findings towards IV superiority. Our study, by focusing strictly on evidence from RCTs, aims to minimize such biases and provide a more robust estimate of the true comparative effectiveness of IO versus IV access as an initial strategy in OHCA.

## 5. Strengths and Limitations

This systematic review and meta-analysis provides the highest certainty of evidence on the comparative efficacy of IO versus IV access in OHCA resuscitation; we included only RCTs, followed PRISMA reporting guidelines, and applied GRADE certainty of evidence assessment. However, our review is limited by the following: First, although we included only RCTs, all trials showed some concerns of performance bias due to the open-label nature of interventions. Second, our meta-analysis did not include safety outcomes due to the heterogeneity or lack of reporting across the included trials. Third, due to the lack of reporting of outcome data, we could not assess the comparative efficacy of administered drugs, such as adrenaline and amiodarone. Finally, the IO route differed among the included studies, with three RCTs permitting either humeral or tibial access [10,12,13] and another investigating humeral access only [11].

## 6. Conclusions

IO and IV vascular accesses showed similar clinical outcomes in OHCA patients, with no difference in ROSC, survival, or neurological recovery. Still, IO access showed a better procedural outcome with increased first-attempt success rates, faster access, and faster drug administration. Accordingly, the choice between IO and IV vascular access should be determined by the specific patient characteristics and capabilities of each local emergency medical service system.

## Figures and Tables

**Table 1:** Summary characteristics of the included RCTs.

Study ID	Study Design	Country	Total Participants	Intraosseous Access Route	Main Inclusion Criteria	Sustained ROSC Definition	Primary Outcome	Follow-Up Duration
Couper et al. 2024 (PARAMEDIC-3) [10]	Multicenter, open-label RCT	UK	6082	Humeral or tibial	Adults (≥18 years) with OHCA (of any cause) and requiring vascular access (during ongoing CPR)	NA	Survival at 30 days	6 months
Ko et al. 2024 (VICTOR) [11]	Multicenter, open-label, cluster RCT	Taiwan	1732	Humeral	Adults (20–80 years) with atraumatic OHCA	ROSC for at least two hours and survival with a favorable neurological outcome	Survival to hospital discharge	Hospital discharge
Reades et al. 2011 [12]	Single-center, open-label RCT	USA	182	Humeral or tibial	Adults (>18 years) with atraumatic OHCA	NA	First-attempt success	NA
Vallentin et al. 2024 (IVIO) [13]	Multicenter, single-blinded RCT	Denmark	1479	Humeral or tibial	Adults (>18 years) with atraumatic OHCA	ROSC with no further use of chest compressions for at least 20 min	Sustained ROSC	6 months

RCT, randomized controlled trial; OHCA, out-of-hospital cardiac arrest; NA, not available; CPR, cardiopulmonary resuscitation; ROSC, return of spontaneous circulation.

**Table 2:** Baseline characteristics of the participants.

Study ID	Number of Patients in Each Group	Age (Years), Mean (SD)		Gender (Male), N. (%)		Bystander CPR, N. (%)				OHCA Cause, N. (%)											OHCA Location, N. (%)				Witness, N. (%)						
		IO	IV	IO	IV	IO	IV	IO	IV	Medical		Traumatic		Drug Overdose		Drowning		Asphyxia		Unknown or Not Recorded		Home		Public		Bystander		EMS		Unwitnessed	
										IO	IV	IO	IV	IO	IV	IO	IV	IO	IV	IO	IV	IO	IV	IO	IV	IO	IV	IO	IV	IO	IV
Couper et al. 2024 (PARAMEDIC-3) [10]		3040	3042	67.8 (16.3)	68.3 (15.9)	1941 (63.8)	1951 (64.1)	2089 (68.7)	2145 (70.5)	2484 (81.7)	2480 (81.5)	48 (1.6)	38 (1.2)	57 (1.9)	67 (2.2)	8 (0.3)	7 (0.2)	86 (2.8)	87 (2.9)	355 (11.7)	362 (11.9)	2392 (78.7)	2422 (79.6)	99 (3.3)	96 (3.2)	1645 (54.1)	1703 (56.0)	194 (6.4)	183 (6.0)	1164 (38.3)	1109 (36.5)
Ko et al. 2024 (VICTOR) [11]		741	991	64.0 (54.0–72.0)	66.0 (56.0–74.0)	521 (70.3)	713 (71.9)	531 (71.7)	701 (70.7)	NA	NA	NA	NA	NA	NA	NA	NA	NA	NA	NA	NA	536 (72.3)	702 (70.8)	187 (25.2)	274 (27.6)	NA	NA	NA	NA	NA	NA
Reades et al. 2011 [12]		115	67	65.7 (2.6)	64.7 (2.2)	77 (65)	42 (63)	NA	NA	NA	NA	NA	NA	NA	NA	NA	NA	NA	NA	NA	NA	NA	NA	NA	NA	NA	NA	NA	NA	NA	NA
Vallentin et al. 2024 (IVIO) [13]		731	748	69 (15)	70 (14)	517 (71)	516 (69)	585 (84)	594 (83)	216 (29)	213 (29)	NA	NA	13 (2)	12 (2)	4 (1)	4 (1)	22 (3)	20 (3)	474 (65)	494 (66)	NA	NA	NA	NA	383 (52)	391 (52)	38 (5)	29 (4)	310 (42)	328 (44)

OHCA, out-of-hospital cardiac arrest; IO, intraosseous; IV, intravenous; N., number; SD, standard deviation; EMS, emergency medical service; NA, not available; CPR, cardiopulmonary resuscitation.

**Table 3:** GRADE evidence profile.

Certainty Assessment							Summary of Findings				
Participants (Studies) Follow-Up	Risk of Bias	Inconsistency	Indirectness	Imprecision	Publication Bias	Overall Certainty of Evidence	Study Event Rates (%)		Relative Effect (95% CI)	Anticipated Absolute Effects	
							With [IV]	With [IO]		Risk with [IV]	Risk Difference with [IO]
Prehospital ROSC											
9277 (3 RCTs)	serious ^a^	not serious	not serious	not serious	none	⨁⨁⨁◯ Moderate ^a^	1548/4774 (32.4%)	1449/4503 (32.2%)	RR 0.97 (0.91 to 1.03)	1548/4774 (32.4%)	10 fewer per 1000 (from 29 fewer to 10 more)
Maintained ROSC											
9250 (3 RCTs)	serious ^a^	not serious	serious ^b^	not serious	none	⨁⨁◯◯ Low ^a,b^	1287/4762 (27.0%)	1108/4488 (24.7%)	RR 0.94 (0.84 to 1.01)	1287/4762 (27.0%)	16 fewer per 1000 (from 43 fewer to 3 more)
Survival to Discharge											
9185 (3 RCTs)	serious ^a^	not serious	not serious	not serious	none	⨁⨁⨁◯ Moderate ^a^	296/4723 (6.3%)	278/4462 (6.2%)	RR 1.00 (0.97 to 1.03)	296/4723 (6.3%)	0 fewer per 1000 (from 2 fewer to 2 more)
30-Day Discharge											
7543 (3 RCTs)	serious ^a^	not serious	not serious	not serious	none	⨁⨁⨁◯ Moderate ^a^	113/3782 (3.0%)	219/3761 (5.8%)	RR 0.98 (0.82 to 1.17)	113/3782 (3.0%)	1 fewer per 1000 (from 5 fewer to 5 more)
Favorable Neurological Recovery											
9185 (3 RCTs)	serious ^a^	not serious	not serious	serious^c^	none	⨁⨁◯◯ Low ^a,c^	222/4723 (4.7%)	218/4462 (4.9%)	RR 1.07 (0.90 to 1.29)	222/4723 (4.7%)	3 more per 1000 (from 5 fewer to 14 more)
Unfavorable Neurological Recovery											
9185 (3 RCTs)	serious ^a^	not serious	not serious	not serious	none	⨁⨁⨁◯ Moderate ^a^	4501/4723 (95.3%)	4244/4462 (95.1%)	RR 1.03 (0.88 to 1.21)	4501/4723 (95.3%)	29 more per 1000 (from 114 fewer to 200 more)
First-Attempt Success											
7743 (3 RCTs)	serious ^a^	not serious	not serious	serious^c^	none	⨁⨁◯◯ Low ^a,c^	2453/3857 (63.6%)	1197/3886 (30.8%)	RR 1.24 (1.19 to 1.29)	2453/3857 (63.6%)	153 more per 1000 (from 121 more to 184 more)
Time Between EMS Arrival and Vascular Access											
7743 (3 RCTs)	serious ^a^	not serious	not serious	serious ^c^	none	⨁⨁◯◯ Low ^a,c^	3857	3886	-	3857	MD 0.24 min lower (0.48 lower to 0.01 lower)
Time Between EMS Arrival and Drug Administration											
7814 (2 RCTs)	serious ^a^	very serious ^d^	not serious	serious ^c^	none	⨁◯◯◯ Very low ^a,c,d^	4033	3781	-	4033	MD 0.38 min lower (0.66 lower to 0.1 lower)

CI: confidence interval; MD: mean difference; RR: risk ratio; explanations: a. All trials had some concerns of performance bias. b. The maintained ROSC definition differed among the included trials. c. A wide confidence interval that does not exclude the appreciable risk of harm or benefit. d. I^2^ > 90%.

## Data Availability

The original contributions presented in this study are included in the article. Further inquiries can be directed to the corresponding author(s).

## Supplementary Materials

The following supporting information can be downloaded at: https://www.mdpi.com/article/10.3390/medsci13020078/s1, Table S1: Search strategy.
