# Supplementary material for: Intraosseous Versus Intravenous Vascular Access in Out-of-Hospital Cardiac Arrest: A Systematic Review and Meta-Analysis of Randomized Controlled Trials
Source: Med Sci (Basel). 2025 Jun 14;13(2):78. doi: 10.3390/medsci13020078 (PMC12195015; doi:10.3390/medsci13020078)
Supplement: Supplementary file 1 [file medsci-13-00078-s001.zip › medsci-3665167-supplementary.pdf]

Supplementary Materials:

Contents:

Tables.

Table S1: Search strategy.

Database	Search Terms	Search Field	Search Results
PubMed	(Intraosseous OR "Intra-osseous") AND (intravenous OR IV) AND ("heart arrest*" OR "cardiac arrest*" OR "cardiopulmonary arrest*" OR "sudden cardiac death" OR SCD OR "OHCA" OR "ventricular tachycardia" OR "ventricular fibrillation" OR "ventricular arrythmia*" OR "pulseless electrical activity" OR PEA OR arrest*)	All Fields	192
Cochrane	(Intraosseous OR "Intra-osseous") AND (intravenous OR IV) AND ("heart arrest*" OR "cardiac arrest*" OR "cardiopulmonary arrest*" OR "sudden cardiac death" OR SCD OR "OHCA" OR "ventricular tachycardia" OR "ventricular fibrillation" OR "ventricular arrythmia*" OR "pulseless electrical activity" OR PEA OR arrest*)	All Text	29
WOS	(Intraosseous OR "Intra-osseous") AND (intravenous OR IV) AND ("heart arrest*" OR "cardiac arrest*" OR "cardiopulmonary arrest*" OR "sudden cardiac death" OR SCD OR "OHCA" OR "ventricular tachycardia" OR "ventricular fibrillation" OR "ventricular arrythmia*" OR "pulseless electrical activity" OR PEA OR arrest*)	All Fields	164
SCOPUS	TITLE-ABS-KEY ((intraosseous OR "Intra-osseous") AND (intravenous OR iv) AND ("heart arrest*" OR "cardiac arrest*" OR "cardiopulmonary arrest*" OR "sudden cardiac death" OR scd OR "OHCA" OR "ventricular tachycardia" OR "ventricular fibrillation" OR "ventricular arrythmia*" OR "pulseless electrical activity" OR pea OR arrest*))	Title, Abstract, Keywords	231

Table S1: Search Strategy.
